# Supplementary material for: Trends in health expectancy at age 60 in Bangladesh from 1996 to 2016
Source: PLoS One. 2022 Nov 23;17(11):e0278101. doi: 10.1371/journal.pone.0278101 (PMC9683622; doi:10.1371/journal.pone.0278101)
Supplement: S1 Text — (DOCX) [file pone.0278101.s001.docx]

**S1 Text. Available data**

**Mortality information**

Reliable nationally representative mortality information for Bangladesh, particularly sex-specific life tables, is available in different formats in three sources:

1. Yearly abridged life tables from 2000 to 2016 are available in World Health Organization’s Global Health Observatory data repository [1].
2. Yearly complete life tables from 1971 to 2100 and 5-yearly abridged life tables from 1970-75 to 2095-2100 are available in World Population Prospects 2019 [2].
3. Yearly abridged life tables for 1991, 2003, and 2006-2018 are available in the Reports on Sample Vital Registration System (SVRS) 2010, 2012-2013 and in the Reports on Bangladesh Sample Vital Statistics (BSVS) 2014-2018 [3].

**Morbidity information**

Reliable nationally representative morbidity information for Bangladesh, particularly disability prevalence and SRH status, is available in three sources as well:

1. Yearly disability prevalence by age-groups and gender for 2009-2018 is available in SVRS 2010, 2012-2013 and in BSVS 2014-2018 [3]. The SVRS 2013 and BSVS 2014-2018 explicitly mention that they have used the following six questions, which are similar to the Washington Group’s short set of disability-related questions, to measure disability:
   1. Problem to see even with spectacles
   2. Problem of hearing even with hearing aids
   3. Problem to rising up
   4. Problem to remember something for sickness
   5. Problem to care self, such as eating, bathing, using toilet and wearing dress
   6. Problem to understand another person and problems of communicating to others and the like.

In a line with SVRS 2013 and BSVS 2014-2018, SVRS 2010-2012 report that they have used six questions to measure disability. However, the types of disability considered in SVRS 2010-2012 are― (i) blind, (ii) night blind, (iii) dumb/deaf, (iv) mentally retarded, (v) Kushtha (i.e., leprosy), (vi) lame, (vii) Otharbo (i.e., frail), (viii) Dhabal (i.e., leucoderma), (ix) Goitre, (x) short in memory, and (xi) others.

1. Nationally representative data on disability prevalence, collected from a total of 55,580 individuals aged 5 years or older, for 2010 are available in Bangladesh’s Household Income and Expenditure Survey (HIES) 2010 [4]. The HIES 2010 adopted and accommodated the Washington Group’s short set of disability-related questions. The short set of six disability-related questions was consistent with the International Classification of Functioning, Disability and Health, a framework for conceptualizing disability developed by the World Health Organization [5]. The six questions were:
   1. Do you have difficulty seeing, even if wearing glasses?
   2. Do you have difficulty hearing, even if wearing a hearing aid?
   3. Do you have difficulty walking or climbing steps?
   4. Do you have difficulty remembering or concentrating?
   5. Do you have difficulty with self-care such as washing all over or dressing, feeding, toileting, etc.? and,
   6. Do you have difficulty communicating, for example, understanding or being understood?

Each question had four response categories: (a) no difficulty, (b) yes, some difficulty, (c) yes, severe difficulty, or (d) yes, cannot perform at all.

1. Nationally representative data on SRH status by age and gender are available in the World Values Survey (WVS) waves of 1994-1998 and 1999-2004. In the two waves, the actual survey years for Bangladesh were 1996 and 2002. The data were collected from 1525 and 1500 individuals aged ≥15 years in 1996 and 2002, respectively [6]. Generally, WVS fields a standardized questionnaire among nationally representative samples of residents (sample size between 1,000 and 3,000 respondents per country). The survey covers the widest array of societies with questions on values, state of health, life satisfaction, and so on. Details on questionnaire wording, fieldwork organization, and data access can be obtained at [www.worldvaluessurvey.org](http://www.worldvaluessurvey.org). In the 1996 WVS, SRH was assessed by a single item: ‘All in all, how would you describe your state of health these days? Would you say it is (a) very good, (b) good, (c) fair, (d) poor, or (e) very poor?’ In the 2002 WVS questionnaire, ‘very poor’ was not included in the above question. We categorized SRH into two groups: ‘good’ and ‘poor’. For the year 1996, ‘very good’, ‘good’ and ‘fair’ were grouped as ‘good’, and ‘poor’ and ‘very poor’ as ‘poor’. In 2002, ‘poor’ state of health alone represents the category ‘poor’, and the other responses were grouped as in 1996 [7].

**References**

1. World Health Organization. Life tables by country - Bangladesh. In: Global Health Observatory data repository [Internet]. World Health Organization; 2020 [cited 6 Apr 2020]. Available: https://apps.who.int/gho/data/view.main.60120?lang=en

2. United Nations, Department of Economic and Social Affairs, Population Division. World Population Prospects 2019. 2019 [cited 6 Apr 2020]. Available: https://population.un.org/wpp/

3. Bangladesh Bureau of Statistics. Bangladesh Sample Vital Statistics. In: Bangladesh Bureau of Statistics [Internet]. 2020 [cited 6 Apr 2020]. Available: http://bbs.portal.gov.bd/site/page/ef4d6756-2685-485a-b707-aa2d96bd4c6c/-

4. Bangladesh Bureau of Statistics. Household Income and Expenditure Survey-2010. New Panama Printing Press, Dhaka: Statistics Division, Ministry of Planning; 2011. Available: http://data.gov.bd/dataset/household-income-and-expenditure-survey-2010

5. Madans JH, Loeb ME, Altman BM. Measuring disability and monitoring the UN Convention on the Rights of Persons with Disabilities: the work of the Washington Group on Disability Statistics. BMC Public Health. 2011;11: S4. doi:10.1186/1471-2458-11-S4-S4

6. Inglehart R, Haerpfer C, Moreno A, Welzel C, Kizilova K, Diez-Medrano J, et al. World Values Survey: All Rounds - Country-Pooled Datafile Version. Madrid: JD Systems Institute; 2014. Available: http://www.worldvaluessurvey.org/WVSDocumentationWVL.jsp

7. Tareque MI, Islam TM, Kawahara K, Sugawa M, Saito Y. Healthy life expectancy and the correlates of self-rated health in an ageing population in Rajshahi district of Bangladesh. Ageing Soc. 2015;35: 1075–1094. doi:10.1017/S0144686X14000130
