# Supplementary material for: Trends in health expectancy at age 60 in Bangladesh from 1996 to 2016
Source: PLoS One. 2022 Nov 23;17(11):e0278101. doi: 10.1371/journal.pone.0278101 (PMC9683622; doi:10.1371/journal.pone.0278101)
Supplement: S1 Table — (DOCX) [file pone.0278101.s002.docx]

**S1 Table.** Poor SRH/ disability prevalence (per 1000) and *annual percent change* in poor SRH/ disability by gender at age 60 from 1996 to 2016

| **Year** | **Poor SRH/ disability rate (per 1000)** | | **Annual percent change in poor SRH/ disability** | |
| --- | --- | --- | --- | --- |
|  | **Men** | **Women** | **Men** | **Women** |
| 1996**^†^** | 263.9 | 285.7 | - | - |
| 2002**^†^** | 169.8 | 0 | -5.9 | -16.7 |
| 2009^‡^ | 48.8 | 51.0 | -10.2 | 728.4 |
| 2010^‡^ | 54.8 | 52.6 | 12.4 | 3.2 |
| 2011^‡^ | 45.8 | 46.1 | -16.5 | -12.5 |
| 2012^‡^ | 47.1 | 46.4 | 2.8 | 0.7 |
| 2013^‡^ | 14.2 | 17.4 | -69.8 | -62.5 |
| 2014^‡^ | 14.9 | 16.3 | 4.5 | -6.3 |
| 2015^‡^ | 16.0 | 15.6 | 7.7 | -4.2 |
| 2016^‡^ | 15.5 | 17.4 | -3.1 | 11.5 |

**Notes:** SRH stands for self-rated health;

^†^ Older adults with poor SRH status; and

^‡^ Older adults with disability.
